# A pilot survey of student perceptions on the benefit of the OSCE and MCQ modalities

**DOI:** 10.3205/zma001197

**Published:** 2018-11-15

**Authors:** Stefan Müller, Utz Settmacher, Ines Koch, Uta Dahmen

**Affiliations:** 1 Jena University Hospital, Department of General, Visceral and Vascular Surgery, Jena, Germany; 2 Jena University Hospital, Department of Gynaecology and Reproductive Medicine, Jena, Germany; 3 Jena University Hospital, Department of General, Visceral and Vascular Surgery, Experimental Transplantation Surgery, Jena, Germany

**Keywords:** medical assessment, OSCE and MCQ modalities, perceptions, students

## Abstract

**Objective: **The objective structured clinical examination (OSCE) has become widely accepted as a form of assessment in medical education. At the same time, the more traditional multiple choice question (MCQ) examinations remain a central modality of student assessment. This pilot survey aimed to investigate students’ perceptions about the benefits of the OSCE and MCQs to yield data supporting the implementation of this assessment strategy into the national medical licensing examination in Germany.

**Methods: **A questionnaire was delivered electronically to 34 German medical schools. Students in years 3-6 were invited to rate 11 items about objectives of good medical assessment. All items were presented for both the OSCE and MCQs using a 5-point Likert Scale (1=strongly disagree to 5=strongly agree). Factor analysis was used to identify underlying components in the ratings. Average scores of items that belonged to a component were computed.

**Results: **Data analysis included 1,082 students from 32 medical schools. For the OSCE, factor analysis revealed two components, which were labelled “educational impact” and “development of clinical competence”. The average scores of items were 3.37 and 3.55, respectively. For the MCQ modality, also two components emerged. These were labelled “perceived weaknesses of MCQs” and “perceived strengths of MCQs” (consisting of items such as “promotes my theoretical knowledge”). The average scores for these components were 1.85 and 3.62.

**Conclusion: **The results of this pilot survey indicate that students consider both OSCE and MCQs as useful assessments for the purposes for which they were designed. The assessment strategy thus appears appropriate and it should be used in the national licensing examination.

## Zusammenfassung

**Zielsetzung: **Objective Structured Clinical Examination (OSCE) hat sich als eine Form der Prüfung in der medizinischen Ausbildung durchgesetzt. Gleichwohl stellen die mehr traditionellen Multiple-Choice-Fragen (MC-Fragen) weiterhin ein zentrales Verfahren bei studentischen Prüfungen dar. Das Ziel dieser Piloterhebung war die Untersuchung der Wahrnehmung von Studierenden über den Nutzen von OSCE und MC-Fragen. Es sollten Daten gewonnen werden, die eine Implementierung dieser Prüfungsstrategie in das medizinische Staatsexamen der Bundesrepublik Deutschland unterstützen.

**Methodik: **Ein Fragebogen wurde elektronisch an 34 medizinische Fakultäten in Deutschland gesandt. Studierende im 3. bis 6. Studienjahr wurden gebeten, 11 Items über Ziele guter medizinischer Prüfungen zu bewerten. Alle Items wurden sowohl für OSCE als auch MC-Fragen auf einer 5-Punkt-Likert-Skala (1=trifft überhaupt nicht zu; 5=trifft vollkommen zu) präsentiert. Eine Faktorenanalyse wurde eingesetzt, um zugrunde liegende Komponenten in den Ratings zu identifizieren. Es wurden Mittelwertindizes aus Items gebildet, die jeweils zu einer Komponente gehörten.

**Ergebnisse:** Die Datenanalyse umfasste 1.082 Studierende von 32 medizinischen Fakultäten. Für das OSCE-Format ergab die Faktorenanalyse zwei Komponenten, die „Bildungswirkung“ und „Entwicklung klinischer Kompetenz“ benannt wurden. Die Mittelwertindizes der Items lagen bei 3,37 bzw. 3,55. Für das MC-Frageformat ergaben sich ebenfalls zwei Komponenten. Diese wurden „empfundene Schwächen von MC-Fragen“ und „empfundene Stärken von MC-Fragen“ (bestehend aus Items wie „fördert mein theoretisches Wissen“) benannt. Die Mittelwertindizes der Komponenten betrugen 1,85 und 3,62.

**Schlussfolgerung: **Die Ergebnisse dieser Piloterhebung zeigen, dass die Studierenden OSCE und MC-Fragen als sinnvolle Prüfungen betrachten, und zwar jeweils für die Ziele, für die sie konzipiert wurden. Die Prüfungsstrategie erscheint daher angemessen und sollte im Staatsexamen angewandt werden.

## 1. Introduction

Assessment is a core aspect of the medical education process. Besides making judgements about a candidate’s competence or performance, assessment influences the curriculum and, more importantly, drives students’ learning. For producing good physicians, medical assessment should adapt to clinical practice and be able to cover the necessary spectrum of competencies [1], [2], [3], [4]. Traditional (written) assessment practices, however, focus on testing students’ knowledge instead of appraising performance related skills [5]. This may explain why medical school graduates are often not adequately prepared for clinical work [6], [7].

The objective structured clinical examination (OSCE) is a performance-based assessment that was developed to appraise a student’s clinical performance. An OSCE typically includes a series of stations where examinees are required to apply their knowledge and skills in simulated settings. At each station, examinees’ performance is rated according to pre-established criteria. The modality has become widely accepted as a form of assessment in many countries [8]. Although OSCEs are now widespread in use, the more traditional multiple choice question (MCQ) examinations continue to play a central role in undergraduate medical education [9], [10].

### 1.1. Medical education in Germany

All 36 medical schools in Germany (i.e. schools founded before 2012) have a six-year undergraduate programme that is based on the statutory provisions of the German licensing regulations for physicians [https://www.gesetze-im-internet.de/_appro_2002/BJNR240500002.html]. The programmes generally comprise three sections: the preclinical years (years 1 and 2), containing basic medical sciences; the clinical years (years 3, 4, and 5), where students are introduced to the various aspects of clinical medicine; and, finally, the clinical internship year (year 6), in which students participate in full-time clinical rotations [11]. At all medical schools, students are required to take in-house exams to register for the national licensing examination.

In conjunction with the amendment of the licensing regulations for physicians [https://www.gesetze-im-internet.de/_appro_2002/BJNR240500002.html] of 2002, the assessment of performance related skills has gained increasing importance. Meanwhile, 34 of the 36 (94.4%) medical schools use OSCE exams as part of their in-house assessment strategy [12]. The use of MCQ examinations, however, still dominates student assessment at German medical schools (own data).

The national medical licensing examination in Germany is a three-part examination. Part I of the examination after year 2 assesses basic medical science subjects. Part II after year 5 is a written assessment to test a student’s clinical knowledge. This part of the examination lasting three days consists of standardised MCQ examinations, which are organised centrally by the Institute for Medical and Pharmaceutical Examination Questions [https://www.impp.de/]. Finally, part III of the examination after year 6 includes a not standardised two-day clinical-practical exam. Recently, it has been shown that this part of the national examination does not sufficiently assess a medical student’s clinical competence [13]. As opposed to other countries, like Switzerland, Canada or the United States [14], [15], [16], the OSCE modality is not yet used in the German medical licensing examination. However, within the framework of the “master plan medical education 2020” [https://www.bmbf.de/de/masterplan-medizinstudium-2020-4024.html], it is intended to incorporate the OSCE assessment into these high-stakes licensure examinations.

#### 1.2. Purpose of the survey

In this pilot survey, we aimed to investigate students’ perceptions about the benefits of the OSCE and MCQ modalities to yield data that support the incorporation of the OSCE into the German medical licensing examination. We focussed on the OSCE and MCQs, as these tools are commonly used methods of assessment at medical schools in Germany, and are, moreover, the key components of other national licensing examinations, for example the Medical Council of Canada Qualifying Examination (MCCQE) and the United States Medical Licensing Examination (USMLE) [15], [16]. We focussed only on the students’ views. The attitudes of the teaching staff were out of scope and thus were not considered in this survey.

## 2. Methods

### 2.1. Survey population

According to data from the Federal Statistical Office [Statistisches Bundesamt], more than 85,000 students (87,863) were enrolled at German medical schools in 2014/15. The majority of them were female students (53,352; 60.7%) [https://www.destatis.de/DE/ZahlenFakten/Indikatoren/LangeReihen/Bildung/lrbil05.html]. With exact information about cohort sizes and assessment schedules at each medical school, we computed the number of students in years 3-6 experienced with the OSCE modality at 34,790.

#### 2.2. Material

An 11-item set was developed. The process of development first involved informal interviews with students from the local medical school. During the interviews, the students were asked to indicate what they expect from good medical assessment in terms of their vocational preparation. The statements obtained were then used to construct the set of items. The set was pretested to ensure that the items were clear and understandable. The complete item set was presented on separate pages for both the OSCE and MCQs, each with the heading “What does … [the modality] do for you?” within a larger questionnaire.^1^ All items were placed on a 5-point Likert Scale (1=strongly disagree, 2=disagree, 3=neither agree nor disagree, 4=agree, and 5=strongly agree). Specifically, the items were:

- A “gives me an understanding of medical care”
- B “demonstrates the practices and principles of medical treatment”
- C “gives me feedback on my performance level”
- D “reveals my strengths in medical practice”
- E “reveals my weaknesses in medical practice”
- F “shows me gaps in my education”
- G “enhances my problem-solving and decision-making abilities”
- H “promotes my theoretical knowledge”
- J “reflects the requirements of the medical profession”
- K “allows me to assess my own ability to work as a medical professional”
- L “helps me with my speciality choice”

Moreover, demographic data on gender, age, academic year, and medical school affiliation were collected at the end of the questionnaire.

#### 2.3. Procedure

The research was performed between February and April 2015. All 34 medical schools using the OSCE modality were solicited to forward a cover letter with the link for accessing the questionnaire to their students in years 3 to 6. Participation in the survey was voluntary and anonymous. No incentives were offered for survey completion. Formal approval from the local research ethics committee was not required.

For ensuring that respondents had personal experience with the OSCE modality, we crosschecked each data set received with the assessment schedule of the relevant medical school. Data were then analysed descriptively. To identify underlying components in the students’ ratings, we entered the data into an exploratory factor analysis with oblique rotation for each of the two assessment methods. We summed up the responses to all items loading highly onto a factor and divided the sums by the number of items to calculate average scores.

## 3. Results

### 3.1. Participants

In all, 1,189 participants completed the questionnaire. One hundred and seven participants were identified who did not have experience with the OSCE modality, gave the same rating for all the items in the set or did not specify their academic year or medical school affiliation (necessary for crosschecking), so their data were excluded from further analysis. Data of 1,082 participants, which corresponds to 3.1% of the target population, from 32 of the 34 medical schools (from two schools there were no respondents at all) were analysed. Of this total, 747 (69.0%) were female students. The age of participants ranged between 19 and 45 years, with a mean of 25.3 and a median of 25 years, covering the full age range of medical students in Germany. The percentages of students in years 4, 5, and 6 were equal (28.0%, 29.8% or 27.0%). The percentage of students in year 3, however, was smaller (15.2%), because of less experience with the OSCE modality.

#### 3.2. Ratings in the 11-item set

Table 1 (Tab. 1) displays the ratings in the 11-item set for the OSCE and MCQs. The OSCE was rated positive (means above 3.50) or weakly positive (means between 3.25 and 3.50) for items B (3.81), A (3.75), F (3.53), E (3.36), D (3.31), and C (3.28). The MCQ modality, by contrast, received positive or weakly positive ratings for items H (4.21), C (3.34), and F (3.32).

Table 2 (Tab. 2) records the item ratings related to the students’ demographic characteristics. The ratings for the items did not vary substantially with respect to gender, age or stage of training (academic year) of participants.

#### 3.3. Factor analysis

Table 3 (Tab. 3) shows the results of the factor analysis including the rotated factor coefficients. The Kaiser-Meyer-Olkin measure of sampling adequacy was 0.90 for the OSCE and 0.87 for the MCQs, respectively, suggesting that data were appropriate for factor analysis. Two factors or components with eigenvalues >1 were found for both modalities. For the OSCE, items E, D, F, and C loaded highly (coefficients >0.60) on factor 1 with loadings of 0.95, 0.84, 0.83 or 0.68. The average score of the four items was 3.37. After inspecting the content of the items, we labelled the component “educational impact”. Items B and A, as well as item H (mean 3.10) loaded highly on factor 2 (factor loadings of 0.80, 0.74, and 0.77, respectively). The average item score was 3.55. Based on the content of the items, we labelled this component “development of clinical competence”. For the MCQ modality, items D, E, K, J, and G (each with a mean below 2.00) had high loadings on factor 1 (factor coefficients of 0.92, 0.89, 0.78, 0.73, and 0.62, respectively), while items H, C, and F loaded highly on factor 2 (coefficients of 0.85, 0.79 or 0.70). The average scores for the two components were 1.85 and 3.62. The components were labelled “perceived weaknesses of MCQs” and “perceived strengths of MCQs”.

## 4. Discussion

This pilot study investigated the benefits of the OSCE and MCQ modalities perceived by students. In the following paragraphs, we discuss the survey findings classified by themes.

Educational impact is considered an important feature of assessment [17], [18]. An assessment, even when it is used for summative purposes, should have a positive effect on students’ future learning by providing feedback to the learners about their strengths and weaknesses. The assessment should also identify areas of weakness in teaching practices or the curriculum so educators can make adjustments. In our study, students rated the items C to F (items that contributed to the component “educational impact”) positive or weakly positive for the OSCE. This indicates that the OSCE modality can serve as a tool to improve both students’ learning and the curriculum, which is in line with previous research [19], [20]. However, it should be pointed out that the provision of detailed feedback to students is difficult to accomplish in a high-stakes licensing OSCE. Nonetheless, the experience gained from taking the assessment as well as the grades achieved can be used by students to enhance their future learning [21].

A practicing physician should be competent in the domains medical knowledge, clinical skills (e.g. taking history from a patient or performing a physical examination), practical procedures (e.g. establishing intravenous access), patient management, communication, and professional behaviour [22], [23]. Helping students develop competencies in the required domains is a major objective of medical education. The item ratings suggest that students perceive the OSCE modality as useful for developing competencies in the domains other than medical knowledge. This conclusion can be drawn from positive ratings for items A and B, vs. a merely mediocre rating for item H (all items that were assigned to the component “development of clinical competence”). Bearing in mind that the OSCE is a performance measure appraising skills and behaviours that are needed in the clinical workplace [8], the present findings are consistent with the OSCE modality’s intended focus.

The greatest perceived strength of the MCQs appears to be in the fostering of the acquisition of knowledge. A strong positive rating for the item “promotes my theoretical knowledge” underpins that. However, our findings indicate that students perceive the MCQ modality to be only suitable to measure lower order cognitive skills (e.g. factual knowledge). This raises the issue of whether medical schools in Germany too often rely on context-free MCQs, which typically consist of discrete questions that aim to test factual knowledge. If so, and in order to include higher order cognitive skills (e.g. processes of problem solving or decision making), it would be advisable to expand the use of context-rich MCQs, in which the questions are directly related to a clinical case presentation [24], [25].

Furthermore, similar to the OSCE, the MCQ modality seems to enable students to evaluate both their own achievements and the content of the curriculum, which is most likely driven by the assessment results. Weakly positive ratings for the items “gives me feedback on my performance level” and “shows me gaps in my education”; vs. low ratings under “reveals my strengths in medical practice” or “reveals my weaknesses in medical practice” suggest this inference.

There are several limitations to this study. First, the study is limited to a relatively small sample representing less than 5% of the target population. This is probably because most medical schools did not inform their students by email, but merely put the cover letter with the link for accessing the questionnaire on their websites. For this reason, a large number of students were likely uninformed about the survey. Second, the study was a pilot survey. The item set presented is not exhaustive and gives an overview only. Despite its limitations, we believe that this survey, the first to investigate the perceptions of students across Germany, provides insight into how and to what extent the OSCE and MCQs are useful assessments. Moreover, our sample was quite representative in terms of gender and age, and we had great diversity regarding the stages of training and medical school affiliations of participants, which make it probable that our results can be generalised to the whole medical student population in Germany.

## 5. Conclusions

The findings of this pilot survey suggest that students consider both assessment modalities, the OSCE and MCQs, to be valuable tools. In summary, the OSCE may have an impact on the educational process and support the development of skills and behaviours required for clinical practice, while the MCQ modality fosters the acquisition of knowledge. Whilst the employment of assessment programmes including a battery of tests will be the most robust strategy to create a global appraisal of a candidate’s knowledge and skills [2], [3], [26], the use of the OSCE and MCQs appears to be an appropriate assessment strategy. This is further evidence of the need to incorporate the OSCE into the German medical licensing examination in addition to the existing MCQs.

## Notes

^1^The questionnaire included items related to various issues. Another manuscript on the students’ learning behaviour when preparing for the OSCE and MCQs has been submitted elsewhere.

## Acknowledgements

The authors wish to acknowledge the deans’ offices of the medical schools, all the participating students, and Prof. Dr. Thomas Kessler and Prof. Dr. Rolf Steyer, both Institute of Psychology at Jena University, for providing support in carrying out this research.

## Competing interests

The authors declare that they have no competing interests.

## Figures and Tables

**Table 1:**
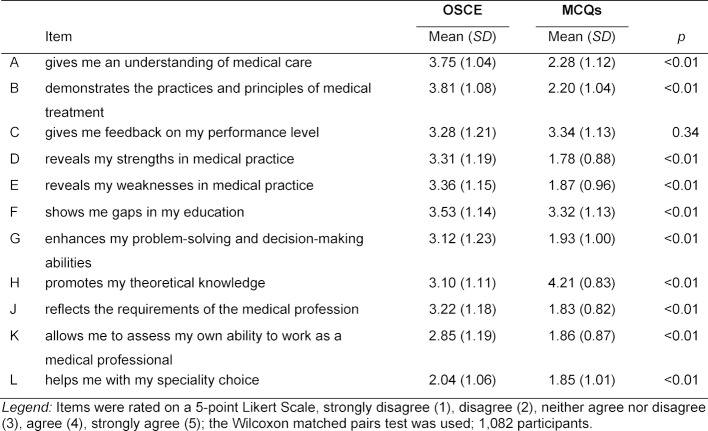
Ratings in the 11-item set for the OSCE and MCQs

**Table 2:**
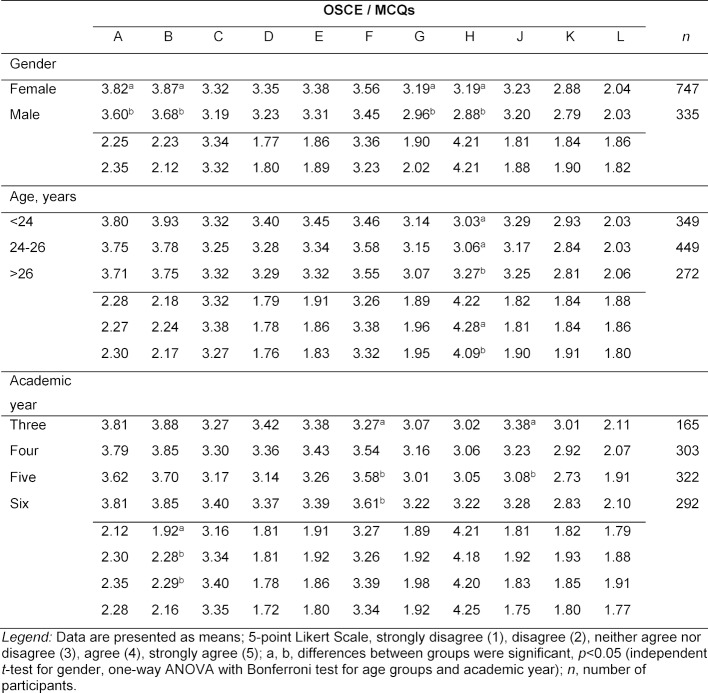
Item ratings related to the students’ demographic characteristics

**Table 3:**
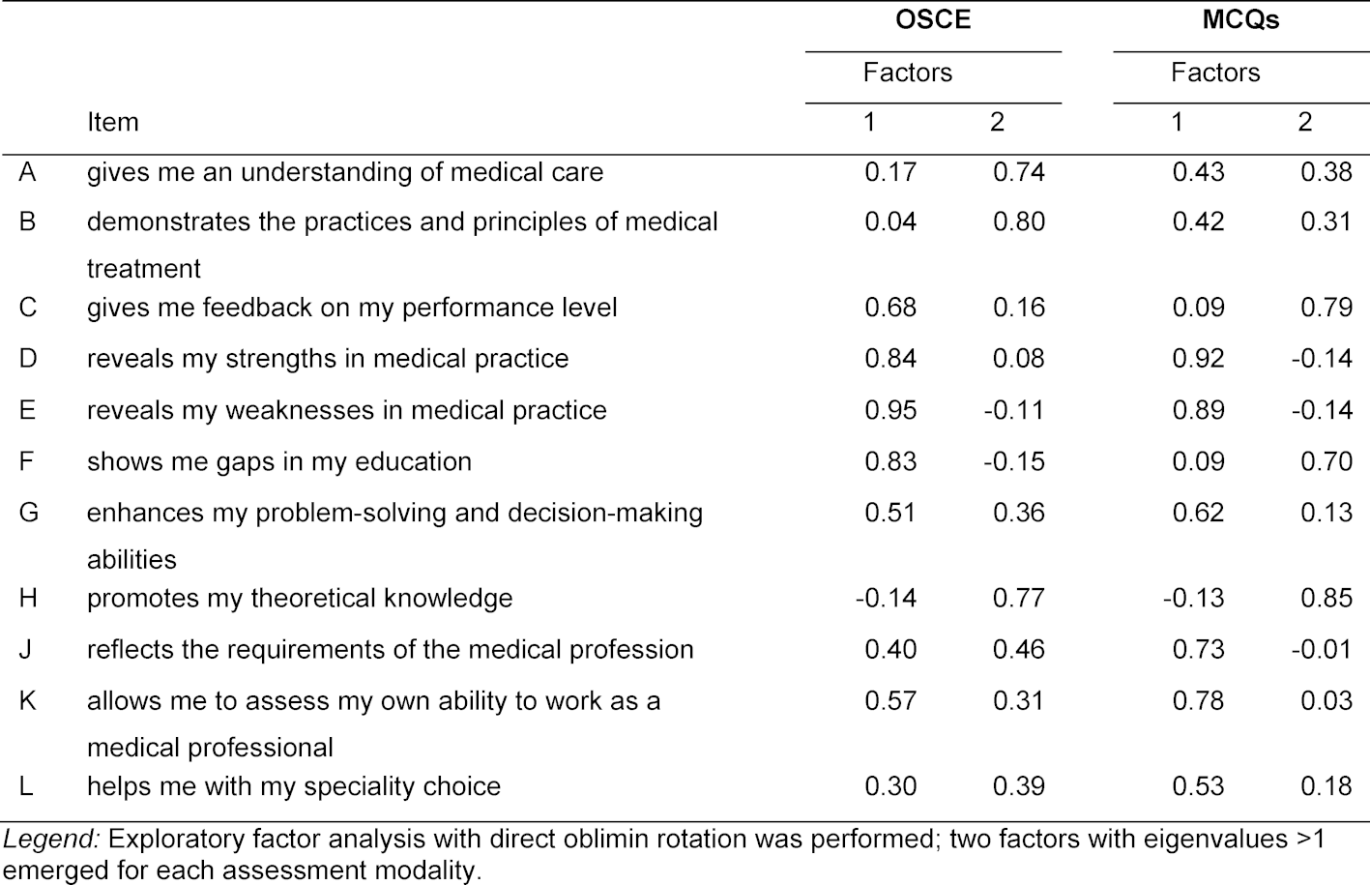
Rotated factor matrix (two factors) for both the OSCE and MCQs
